# Short-Term Effects of Two COX-2 Selective Non-Steroidal Anti-Inflammatory Drugs on the Release of Growth Factors and Cytokines from Canine Platelet-Rich Gel Supernatants

**DOI:** 10.3390/gels10060396

**Published:** 2024-06-12

**Authors:** Julián Ospina, Jorge U. Carmona, Catalina López

**Affiliations:** 1 Grupo de Investigación Patología Clínica Veterinaria, Departamento de Salud Animal, Universidad de Caldas, Calle 65 No 26-10, Manizales 170004, Colombia; julianospina55@gmail.com; 2 Grupo de Investigación Terapia Regenerativa, Departamento de Salud Animal, Universidad de Caldas, Calle 65 No 26-10, Manizales 170004, Colombia

**Keywords:** platelet-rich plasma, platelet-rich gel, dog, firocoxib, carprofen, osteoarthritis, growth factors, cytokines

## Abstract

(1) Background: There is a lack of knowledge about how a single dose of COX-2 selective non-steroidal anti-inflammatory drugs (NSAIDs) might affect the release of growth factors (GFs) and cytokines from canine platelet-rich gels (PRGs) and other hemocomponents. (2) Methods: A crossover study was conducted in six adult mongrel dogs. Animals were randomized to receive a single dose of either carprofen or firocoxib. PRG, temperature-induced platelet lysate (TIPL), chemically induced PL (CIPL), and plasma hemocomponents were obtained from each dog before (1 h) and after (6 h) the treatments. Platelet and leukocyte counts and determination of the concentrations of platelet-derived growth factor-BB, (PDGF-BB), transforming growth factor beta-1 (TGF-β_1_), interleukin 1 beta (IL-1β), tumor necrosis factor-alpha (TNF-α) and IL-10 concentrations were assayed by ELISA in all hemocomponents. (3) Results: Both platelet and leukocyte counts and PDGF-BB concentrations were not affected by NSAIDs and time. Total TGF-β_1_ concentrations were not affected by NSAIDs; however, the release of this GF was increased in PRG supernatants (PRGS) at 6 h. IL-1β and TNF-α concentrations were significantly (*p* < 0.001) lower in both firocoxib PRGS and plasma at 6 h, respectively. IL-10 concentrations were significantly (*p* < 0.001) lower at 6 h in all hemocomponents treated with both NSAIDs. (4) Conclusions: The clinical implications of our findings could indicate that these drugs should be withdrawn from patients to allow their clearance before the clinical use of PRP/PRG. On the other hand, the prophylactic use of NSAIDs to avoid the inflammatory reactions that some patients might have after PRP/PRG treatment should be performed only in those animals with severe reactive inflammation to the treatment.

## 1. Introduction

Platelet-rich plasma (PRP), a hemocomponent mainly obtained from anticoagulated whole blood by centrifugation processes, is becoming one of the most popular biomaterials for regenerative medicine goals in several medical fields, including orthopedics, ophthalmology, aesthetic surgery, and neurosurgery, among others [1,2,3,4,5,6,7].

The rationale for the use of PRP stems from the fact that once this biodrug comes into contact with chemical activators, such as calcium salts or thrombin, or with collagen from the injected tissues, it polymerizes into a gel (platelet-rich gel (PRG)) that gradually releases growth factors (GFs) and cytokines, allowing cell traffic and stem cell migration, and differentiation, among other physiological actions that produce modulation of the inflammation and, lately, regeneration of the affected tissues [8,9].

Currently, PRP is one of the most commonly used orthobiologic products employed for the treatment of musculoskeletal disorders in humans [10,11,12] and animals, such as dogs [13,14,15] and horses [15,16,17]. In general, this biologic has been successfully used to treat osteoarthritis (OA), tendinopathies, and neuropathies [13,16,17].

In some cases, PRP is used as a second-line treatment for the aforementioned conditions, after common symptomatic treatments, such as non-steroidal anti-inflammatory drugs (NSAIDs), corticosteroids, and physical therapy, have failed. However, there is a lack of information on how selective cyclooxygenase-2 (COX-2) NSAIDs might affect the release of GFs (i.e., transforming growth factor-beta 1 (TGF-β_1_) and platelet-derived growth factor isoform BB (PDGF-BB) and cytokines (interleukin 1 beta (IL-1β), tumor necrosis factor-alpha (TNF-α), and IL-10) from platelet-rich gels (PRGs) of patients who may require targeted treatment with PRP. However, it is known that nonselective COX-2 NSAIDs such as aspirin [18] and naproxen [19] can reduce the release of PDGF and vascular endothelial growth factor (VEGF) when multiple doses are used. In addition, the release of growth factors and cytokines from PRP was not affected by a single dose of ketoprofen and flunixin meglumine in horses [20].

On the other hand, some patients may have an inflammatory and painful reaction after receiving a PRP injection. Therefore, some physicians may recommend the prophylactic use of NSAIDs to prevent inflammatory reactions when PRP is used in certain patients or in those who have shown an inflammatory reaction to an initial dose of PRP and require additional treatments with this orthobiologic.

Dogs are animals with similar musculoskeletal pathologies to humans, such as OA [21,22]. Furthermore, both species may have similar pharmacokinetic patterns to NSAIDs [23]. Thus, dogs can be used as a model to study the early effect of COX-2-selective NSAIDs on the mediator release from PRGs. On the other hand, according to the literature review, there are no published data about the effect of NSAIDs on GF and cytokine release from canine PRG.

The aim of this study was to determine the early effects (6 h) of two COX-2-selective NSAIDs, firocoxib and carprofen, on the release of TGF-β_1_, PDGF-BB, IL-1β, TNF-α, and IL-10 from canine PRGs.

## 2. Results and Discussion

### 2.1. Platelet and Leukocyte Concentrations in Whole Blood and Hemocomponents

**Figure 1:**
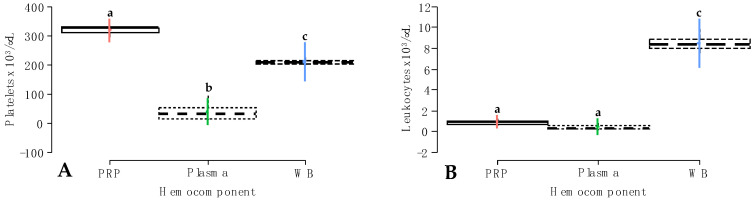
Box plots showing the means and their 95% confidence intervals (CIs) for (**A**) platelet and; (**B**) leukocyte concentrations according to the hemocomponent factor. ^a–c^ = different lowercase letters denote significant differences (*p* < 0.001) for the variables evaluated by the Tukey test between hemocomponents. PRP = platelet-rich plasma; WB = whole blood.

Platelet and leukocyte concentrations were significantly affected by the hemocomponent factor (Figure 1A,B), whereas the other fixed factors and their interactions did not affect the models (Table 1). Platelet counts were significantly (*p* < 0.001) increased in PRP, followed by whole blood and plasma. In general, there were significant differences in this variable between hemocomponents (Figure 1A). On the other hand, leukocyte concentration was significantly higher in whole blood when compared to PRP and plasma. There was no difference between these latter hemocomponents (Figure 1B).

There are several types of PRP that may differ in the concentrations of mediators (GFs and cytokines) released after activation and subsequent gelation. Although several classification systems have been proposed to organize the vast taxonomy of PRP products, in general, PRP preparations can be classified as pure PRP (P-PRP) and platelet- and leukocyte-rich PRP (L-PRP). Technically, a P-PRP product has a negligible concentration of leukocytes with a low to moderate concentration of platelets (from one to three times the platelet count of whole blood), whereas L-PRP has detectable to higher concentrations of leukocytes and higher concentrations of platelets (from three to five times the platelet count of whole blood) [24,25]. According to the above classification, the PRP product evaluated in the present study can be classified as P-PRP.

Notably, platelet and leukocyte counts in whole blood, PRP, and plasma were not affected by treatment and time factors. Our results are consistent with previous studies of the same nature in humans [18,19] and horses [20], where significant changes were not associated with the type of NSAID or the time.

### 2.2. Growth Factor Release from Hemocomponents

**Figure 2:**
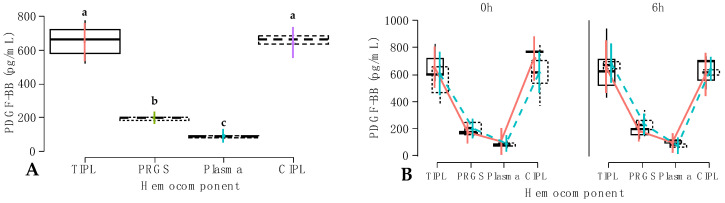
Box plots showing the means and their 95% CIs for (**A**) PDGF-BB concentrations (pg/mL) by hemocomponent factor; (**B**) PDGF-BB concentrations (pg/mL) according to the interaction between hemocomponent, treatment, and time factors. 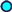 Carprofen; 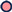 Firocoxib. ^a–c^ = different lowercase letters denote significant differences (*p* < 0.001) for the evaluated variables by the Tukey test. TIPL = temperature-induced platelet lysate; PRGS = platelet-rich gel supernatant; CIPL = chemically induced platelet lysate. Other acronyms are the same as in Table 2.

In model 3, only PDGF concentrations were affected by the hemocomponent effect, whereas the other fixed factors and their interactions did not influence the concentrations of this GF (Table 2). Significantly higher PDGF-BB concentrations were observed in temperature-induced platelet lysates (TIPLs) and chemically induced platelet lysates (CIPLs) compared to PRGS and plasma. Both TIPL and CIPL PDGF-BB concentrations were similar, whereas the concentrations for this GF were significantly different between PRGS and plasma (Figure 2A).

Of note, this growth factor was not affected by the NSAID type (treatment effect) and time effect, although carprofen showed a nonsignificant trend of higher values at 6 h (Figure 2B).

**Figure 3:**
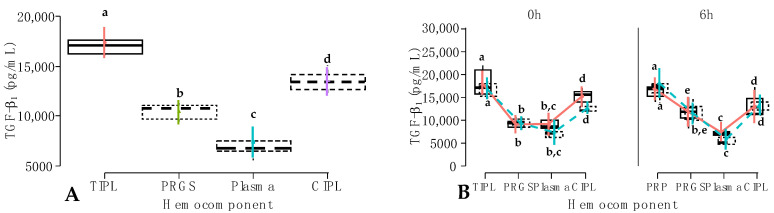
Boxplots showing the means and their 95% CIs for (**A**) TGF-β_1_ concentration (pg/mL)) by the hemocomponent factor; (**B**) TGF-β_1_ concentration (pg/mL) according to the interaction between hemocomponents, treatment, and time. 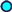 Carprofen; 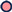 Firocoxib. ^a–e^ = different lowercase letters denote significant differences (*p* < 0.001) for the evaluated variables by the Tukey test. Acronyms are the same as in Figure 2 and Table 2.

In model 4, TGF-β_1_ concentrations were affected by the hemocomponent factor and the interaction between hemocomponents, treatment, and time factors (Table 2). Concentrations for this GF were significantly (*p* < 0.001) different for each hemocomponent, with higher concentrations for TIPL, followed by CIPL, PRGS, and plasma (Figure 3A).

When Tukey contrasts were performed to detect potential differences between the interaction of hemocomponents, treatment, and time factors, it was observed that TGF-β_1_ concentrations were significantly different between carprofen and firocoxib TIPL groups and the firocoxib CIPL group at 1 h (*p* < 0.001), between the firocoxib TIPL group and firocoxib CIPL group at 6 h (*p* < 0.001), and between the firocoxib TIPL group and the firocoxib CIPL group at 6 h (*p* < 0.001). Furthermore, carprofen PRGS at 6 h presented higher TGF-β_1_ concentrations than both carprofen and firocoxib PRGS groups at 1 h (Figure 3B).

In the present study, we evaluated the effect of two COX-2 selective AINEs (carprofen and firocoxib) commonly used in canine practice on PDGF-BB, TGF-β_1_, IL-1β, TNF-α, and IL-10 concentrations released from four hemocomponents (TIPL, CIPL, PRGs and plasma), of which one (CIPL) was used as a positive control for GF enrichment and another for GF depletion (plasma) [26]. It is necessary to clarify that, technically, the term “hemocomponent” could refer to a product obtained from blood fractionation by using physical processes, such as centrifugation, filtration, or apheresis [27].

Currently, both TIPL and PRP/PRG are hemocomponents mainly used for cell culture and therapeutic purposes, respectively. However, the major difference between TIPL and PRP/PRG is that this latter hemocomponent has living cells at the time of the injection [28], whereas the TIPL is a rich source of mediators without living cells and with varying degrees of cell debris [29].

PDGF-BB and TGF-β_1_ were selected in the present study because these mediators are mainly found in platelets. They are directly involved in the processes of inflammation resolution, chemotaxis, and neovascularization [30,31]. All of these actions are necessary to induce wound healing and subsequent regeneration. PDGF-BB has important chemotactic effects on stem cells and is a potent angiogenic growth factor [32]; whereas TGF-β_1_ is a powerful anti-inflammatory mediator with anabolic effects on fibroblasts, which in turn induce extracellular matrix deposition [33].

In the present study, PDGF-BB concentrations were significantly higher in TIPL and CIPL compared to PRGS and plasma, and no effect was produced by the treatment and time factors. The fact that PDGF concentrations were similar in TIPL and CIPL may indicate that this cytokine was not denatured by the effect of temperature changes or by the effect of Triton X-100 detergent. On the other hand, our results also indicate that after 3 h of activation with calcium gluconate, PRG clots had released at least 50% of the total PDGF-BB contained in this hemocomponent in comparison to TIPL and CIPL. This fact demonstrates that PRG is an active biomaterial that could control the gradual release of PDGF when used in different clinical applications.

Regarding the behavior of PDGF release in hemocomponents of our study, it indicates that in the short term, a single therapeutic dose of carprofen or firocoxib did not affect the concentration of this anabolic mediator in the studied hemodynamic components. At this point, it is necessary to know if the chronic consumption of these drugs can reduce the concentration of this GF, as it has been observed in humans treated with aspirin [18] or naproxen [18].

In terms of the influence of the hemocomponent factor, TGF-β_1_ concentrations from the highest to lowest values were obtained in TIPL, followed by CIPL, PRGS, and plasma. At this point, it is necessary to clarify that the gold standard (anionic detergent) is commonly used to demonstrate GF enrichment [26] produced significantly lower TGF-β_1_ concentrations in CIPLs than in TIPLs. This finding was similar to a previous bovine blood study, in which the concentrations of this GF were significantly lower in CIPLs when compared to TIPLs [29], suggesting that this substance may cause selective chemical denaturation of some GFs, since PDGF-BB concentrations in the present study were similar between CIPLs and TIPLs.

Regarding the effects of the interaction between hemocomponents, treatment, and time factors, the most important finding was that TGF-β_1_ concentrations of the carprofen PRGS group at 6 h were higher than those concentrations observed for both carprofen and firocoxib PRGS groups at 1 h. This finding could indicate that a single therapeutic dose of carprofen could selectively accelerate the release of this GF from platelets compared to PDGF-BB. This finding contradicts the results observed in a human study where the chronic aspirin low-dose (81 mg) administration decreased the ability to release TGF-β_1_ from PRGS [18]. Furthermore, our findings differ from those obtained in horses where a single dose of either phenylbutazone or firocoxib after 6 h did not induce changes in the platelet’s ability to release this GF [20].

### 2.3. Cytokine Release from Hemocomponents

In model 5, IL-1β concentrations were significantly affected by the interaction between hemocomponent and time factors (*p* = 0.019) and between hemocomponent, treatment and time factors (*p* < 0.001). Notably, the concentrations for this proinflammatory cytokine were not affected by the individual effect of the fixed factors (Table 3).

**Figure 4:**
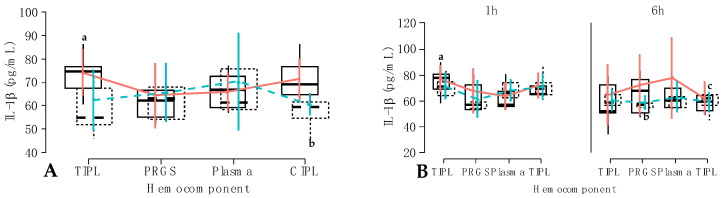
Box plots showing the means and their 95% CIs for (**A**) IL-1β concentrations (pg/mL) by the interaction between hemocomponent and time factors. 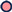 One h; 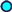 6 h; (**B**) IL-1β concentrations (pg/mL) according to the interaction between hemocomponents, treatment, and time. 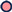 Carprofen; 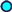 Firocoxib. ^a–c^ = different lowercase letters denote significant differences (*p* < 0.001) for the evaluated variables by the Tukey test. Acronyms are the same as in Figure 2 and Table 3.

Regarding this last interaction, there were significant differences between IL-1β concentrations from TIPLs (*p* < 0.001), plasma (*p* = 0.047), and CIPLs of carprofen-treated dogs (*p* < 0.001) at 1 h when compared to plasma of firocoxib-treated dogs at 6 h (Figure 4A).

IL-1β concentrations were significantly (*p* < 0.001) different between the TIPL group at 1 h and the CIPL group at 6 h (Figure 4B). On the other hand, when the interactions between hemocomponents, treatment, and time were evaluated, significant differences were found between IL-1β concentrations of TIPLs from the carprofen group at 1 h and PRGS (*p* = 0.004) and CIPLs (*p* = 0.004) from the firocoxib group at 6 h (Figure 4B).

**Figure 5:**
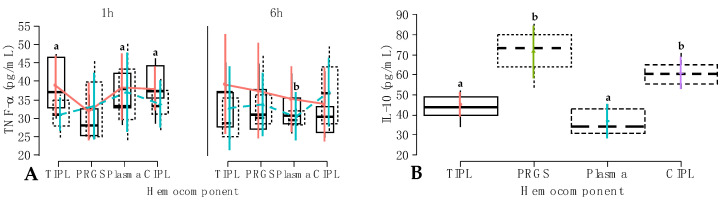
Box plots showing the means and their 95% CIs for (**A**) tumor necrosis factor-alpha (TNF-α) concentrations (pg/mL) by the interaction between hemocomponent, treatment, and time factors. 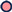 Carprofen; 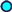 Firocoxib; (**B**) IL-10 concentrations (pg/mL) according to the hemocomponent factor. ^a,b^ = different lowercase letters denote significant differences (*p* < 0.001) for the evaluated variables by the Tukey test. Acronyms are the same as in Figure 2 and Table 3 and Table 4.

In model 6, TNF-α concentrations were not affected by the individual fixed factors. However, there was a significant (*p* < 0.001) interaction between hemocomponent, treatment, and time factors (Table 3, Figure 5A). IL-1β and TNF-α were selected in the present study because they are cytokines involved in the high pathways of the inflammatory process. Both cytokines have been implicated as key mediators in chronic musculoskeletal degenerative/inflammatory diseases such as osteoarthritis, teno-desmopathies, and spondylopathies, among others [34,35,36].

In the present study, IL-1β concentrations of CIPLs at 6 h were significantly lower compared to TIPL at 1 h. This finding could be considered incidental when evaluating the effects of the interaction between hemocomponent and time factors. However, when the treatment factor was added to the above interaction, it was found that firocoxib at 6 h induced lower concentrations of this cytokine in PRPs compared to carprofen TIPL at 1 h. A similar trend was observed in PRP from firocoxib-treated horses at 6 h, although this fact was not statistically significant in this study [20].

TNF-α concentrations in the firocoxib plasma group at 6 h were significantly (*p* < 0.001) lowest compared to carprofen TIPLs, plasma, and CIPLs at 1 h (Figure 5A). However, both carprofen and firocoxib did not affect the release of this inflammatory cytokine in PRGS at any time point. Taken together, these results suggest that the systemic production of this inflammatory cytokine is not exclusively related to circulating leukocytes and that firocoxib may have a more potent systemic anti-inflammatory effect than carprofen in dogs [37,38], not only related to COX-2 inhibition but also to the blockade of higher inflammatory pathways directly related to TNF-α production [39].

In model 7, IL-10 concentrations were significantly influenced by the fixed hemocomponent (*p* < 0.001) and time (*p* < 0.001) factors, whereas the interactions between hemocomponent and treatment factors (*p* = 0.046), hemocomponents and time (*p* = 0.001), and hemocomponent, treatment, and time factors (*p* < 0.001) significantly influenced the concentrations of this anti-inflammatory mediator (Table 4). IL-10 concentrations were significantly (*p* < 0.001) higher in PRGS and CIPL hemocomponents compared to TIPL and plasma hemocomponents. On the other hand, there were no differences in the concentrations of this cytokine between PRGS and CIPL hemocomponents and TIPL and plasma hemocomponents (Figure 5B).

**Figure 6:**
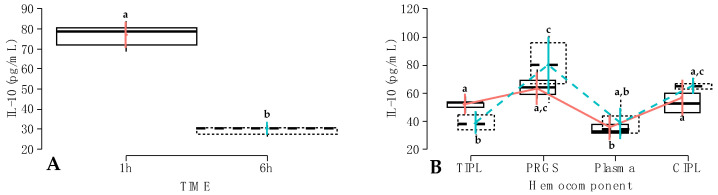
Box plots showing the means and their 95% CIs for (**A**) IL-10 concentrations (pg/mL) by the time factor; (**B**) IL-10 concentrations (pg/mL) according to the interaction between hemocomponent and treatment factors. 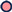 Carprofen; 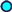 Firocoxib ^a–c^ = different lowercase letters denote significant differences (*p* < 0.001) for the evaluated variables by the Tukey test.

When the effect of the time factor was evaluated, significant differences were observed between IL-10 concentrations measured at 1 h compared to 6 h (*p* < 0.001). In particular, total IL-10 concentrations decreased dramatically from 1 h to 6 h (Figure 6A).

Regarding the interaction between hemocomponent and treatment factors, total IL-10 concentrations were significantly higher in carprofen TIPLs compared to firocoxib TIPLs (*p* < 0.001). Both carprofen and firocoxib plasma groups had significantly (*p* < 0.001) lower concentrations of IL-10 compared to both carprofen and firocoxib PRGS groups. On the other hand, firocoxib CIPL had significantly higher IL-10 concentrations than both firocoxib TIPL and plasma (Figure 6B).

**Figure 7:**
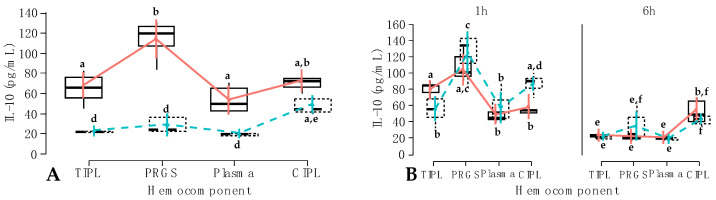
Box plots showing the means and their 95% CIs for (**A**) IL-10 concentrations (pg/mL) by the interaction between hemocomponent and time factors. One h; 6 h; (**B**) IL-10 concentrations (pg/mL) according to the interaction between hemocomponent, treatment, and time factors. 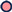 Carprofen; 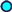 Firocoxib. ^a–f^ = different lowercase letters denote significant differences (*p* < 0.001) for the evaluated variables by the Tukey test.

Regarding the interaction between hemocomponent and time factors, IL-10 concentrations in each hemocomponent group were significantly affected by the time factor. In general, IL-10 concentrations at 1 h were significantly (*p* < 0.001) higher for each hemocomponent group compared to their similar groups at 6 h. The concentrations for this anti-inflammatory cytokine at 1 h were significantly (*p* < 0.001) higher in PRGS compared to the rest of the evaluated hemocomponents. Notably, there were no differences in IL-10 levels between TIPL, plasma and CIPL groups at 1 h (Figure 7A). Furthermore, at 6 h, IL-10 concentrations were similar between the TIPL, PRGS, and plasma groups, whereas the concentrations for this mediator were significantly (*p* < 0.001) higher in the CIPL group compared to the rest of the evaluated hemocomponents (Figure 7A).

When the interaction between hemocomponent, treatment, and time factors was evaluated, several significant differences were observed. First of all, the firocoxib-PRGS group presented the highest IL-10 concentrations, which differed significantly (*p* < 0.001) from the TIPL, plasma, and CIPL groups of both NSAIDs at 1 h. At 1 h, significant differences were also observed for the concentrations of this cytokine between carprofen and firocoxib TIPLs (*p* < 0.001), firocoxib TIPLs and both carprofen and firocoxib PRGSs (*p* < 0.001), and carprofen CIPLs and firocoxib CIPLs (*p* < 0.001). At 6 h, IL-10 concentrations significantly decreased in the evaluated hemocomponents in comparison to the cytokine concentrations obtained for the same groups at 1 h. At this time point (6 h), both carprofen and firocoxib CIPLs presented significantly (*p* < 0.001) highest concentrations of this mediator when compared to carprofen and firocoxib TIPLs and plasma, and carprofen PRGSs. Of note, IL-10 concentrations from firocoxib PRGSs were similar to both carprofen and firocoxib CIPLs (Figure 7B).

The IL-10 assay was included in this study because this anti-inflammatory cytokine prevents cartilage degradation by upregulating the synthesis of extracellular matrix components such as aggrecan and type II collagen. It also downregulates TNF-α and IL-1β and matrix metalloproteinases [40]. The results of this hemocomponent factor effect study differed from those of Gallego et al. [41], who found no significant differences in IL-10 levels between PRP and plasma, although in a human PRP study, differences were found in the levels of this cytokine between PRP and serum [42]. However, the most important finding related to the effect of the hemocomponent factor in the present study was the fact that IL-10 concentrations were better preserved in CI-PLs than in TIPLs, which could indicate that extreme temperature changes may denature this cytokine [29].

In general, a significant effect of time factor on IL-10 concentrations was observed in the present study. This effect was also observed when the interactions between hemocomponent and time factors and hemocomponent, treatment, and time factors were evaluated. Taking all these results together and considering the aims of this study, it can be asserted that both carprofen and firocoxib can produce a strong depression of the systemic production of this anti-inflammatory cytokine in dogs. These results differ from those reported in the PRP of horses treated with firocoxib and other NSAIDs, where IL-10 concentrations were not affected at 6 h after a single therapeutic dose of these drugs [20]. On the other hand, other studies in different animal species have found that carprofen does not affect the expression of IL-10 over time [43], whereas firocoxib reduces the expression of this cytokine after its application [39].

## 3. Conclusions

A single therapeutic dose of either carprofen or firocoxib did not affect PDGF-BB concentrations over time; however, these drugs can accelerate the release of TGF-β_1_ from canine PRGs at 6 h without affecting total levels of this mediator. Firocoxib decreased the release of IL-1β from canine PRG and decreased TNF-α plasma concentrations at 6 h. Both carprofen and firocoxib decreased the production and release of IL-10 from canine PRG. These results may indicate that these COX-2 selective NSAIDs, not only decrease the production of prostaglandins, but also produce a modification of the high pathways of the inflammatory process.

The clinical implications of our findings warn that it is advisable to withdraw the administration of these drugs with sufficient time to safely obtain PRP. On the other hand, the prophylactic use of NSAIDs to avoid the inflammatory reactions that some patients might have after PRP/PRG treatment should be performed only in those animals with severe reactive inflammation to the treatment. Further studies are needed to know the chronic effect of these drugs on GF and cytokine release from PRGS and other related hemocomponents but also more studies on early or acute effects should be ensured.

## 4. Materials and Methods

This prospective, crossover, randomized, controlled study was approved by the Animal Experimentation Committee of the Universidad de Caldas, Manizales, Colombia (Project code: PRY34-2023, date of approval 19 June 2023). The owners of the dogs enrolled were informed of the nature of the study and signed an informed consent form.

### 4.1. Animals

Six clinically healthy mongrel dogs (3 males and 3 females) with a mean age of 3 years (range: 1–4 years) and a mean weight of 23 kg (±7 kg) were included. The health of each animal was assessed by physical examination, complete blood count (CBC), urinalysis, and renal and hepatic clinical chemistry panels.

### 4.2. Study Design, Blood Procurement, and Hemocomponent Processing

Whole blood was collected aseptically from jugular veins using a butterfly catheter coupled to vacuum tubes containing sodium citrate as an anticoagulant. A total of nine 3.6 mL tubes were collected for each dog. Anticoagulated blood from one tube was used for automated CBC (Celltac α MEK-6450. Nihon Kohden, Tokyo, Japan) and plasma procurement by centrifugation of this blood tube at 5000 g/6 m.

Briefly, PRP was obtained by centrifuging the blood tubes at 191 g/6 min. After the anticoagulated blood was separated into three layers (red blood cells (packed cell volume (PCV), buffy coat, and plasma), 75% of the supernatant plasma adjacent to the buffy coat was gently aspirated with a spinal needle 18 G pair into a 10 mL syringe and then packed into a 10 mL sterile PET tube. The plasma in this tube was considered PRP [44]. This hemocomponent was also evaluated for automated CBC.

Blood samples for CBC and the hemocomponent collection were obtained 1 h before and 6 h after the dogs received a randomized oral dose of either 5 mg/kg firocoxib (Previcox, Boehringer Ingelheim, Ingelheim, Germany) or 4.4 mg/kg carprofen (Rimadyl, Zoe-tis, Parsippany, NJ, USA). After a three-week washout period, the dogs that had received one specific oral COX-2 selective NSAID were treated with the other evaluated drug, and the hemocomponents were again obtained 1 h before and 6 h after treatment.

The hemocomponents evaluated in the study at 1 h and 6 h were plasma, PRG supernatant (PRGS) obtained from PRP activated with calcium gluconate solution (9.3 mg/mL) (Ropsohn Therapeutics Ltd.a^®^, Bogotá, Colombia) in a 10:1 ratio, temperature-induced platelet lysate (TIPL), and chemically induced platelet lysate (CIPL). Note that platelet lysates were obtained from the same PRPs. TIPL was obtained from a PRP sample frozen at −80 °C for 30 min and thawed at room temperature for 30 min. CIPLs were obtained from a PRP sample mixed in a 1:10 ratio with a nonionic surfactant detergent (Triton X100, PanReacAppliChem, Barcelona, Spain). 

### 4.3. Growth Factor and Cytokine Assessment in Hemocomponents

TGF-β_1_, PDGF-BB, IL-1β, TNF-α, and IL-10 concentrations were measured in PRGS, TIPL, CIPL, and plasma obtained from dogs treated with the two COX-2 selective NSAIDs at 1 and 6 h. These polypeptides were assayed by ELISA in duplicate using development kits from R&D Systems (Minneapolis, MN, USA). TGF-β_1_ (Human TGF-β1 DuoSet, DY240E) and PDGF-BB (Human PDGF-BB DuoSet, DY220) were determined using human antibodies because of the high sequence homology between dog and human [45,46]. On the other hand, similar ELISA kits have been used and validated for the same purposes in other canine [41,44] and equine [47,48] PRP studies. 

IL-1β (Canine IL-1 Beta/IL-1F2 DuoSet, DY3747), TNF-α (Canine TNF-alpha DuoSet, DY1507), and IL-10 (Canine IL-10 DuoSet, DY735) were tested using species-specific canine antibodies for these cytokines. The standards provided with each ELISA kit were used to construct each standard curve according to the manufacturer’s instructions. Absorbance readings were performed at 450 nm [41,44].

### 4.4. Statistical Analysis

Data were analyzed using the free statistical software JASP (JASP (Intel 0.18.3), University of Amsterdam, The Netherlands). Generalized linear mixed models (GLMMs) were performed to evaluate the effect of the fixed factors: hemocomponents (4 levels), treatment (2 levels), and time (2 levels) on the response variables. The interaction between the fixed factors was also included in each model. GLMMs were performed using Gaussian distributions family with identity as the link function. In addition, dog ID was declared as a random factor in all models. When models showed significant differences, a Tuckey post-hoc test was performed to contrast means by fixed factors and their interactions. A *p* < 0.05 was accepted as significant for all tests performed.

Sample size and power were calculated after obtaining the first three means and their respective standard deviations of each cellular and biochemical parameter for each NSAID evaluated by considering a β value of 0.8 and a α value of 0.05. Accordingly, the n calculated for each group was a minimum of 5 dogs per experimental group. However, one additional dog was included to balance the design and improve the final statistical power of the study.

In addition, the effect of the gender fixed factor (two levels: male and female) was not included in the final modeling of the study because it did not affect the preliminary statistical models conducted in this research. 

## Figures and Tables

**Table 1:** Generalized linear mixed models (GLMMs) evaluating the effect of the fixed factors and their interactions on platelet (model 1), and leukocyte concentration (model 2).

GLMM Number	Fixed Effect	*p*-Value
	Intercept	<0.001
	Hemocomponent (HC)	<0.001
	Treatment	0.432
1	Time	0.552
	HC × Treatment	0.961
	HC × Time	0.816
	Treatment × Time	0.826
	HC × Treatment × Time	0.736
	Intercept	<0.001
	HC	<0.001
	Treatment	0.198
2	Time	0.269
	HC × Treatment	0.242
	HC × Time	0.156
	Treatment × Time	0.201
	HC × Treatment × Time	0.549

**Table 2:** GLMMs evaluating the effect of the fixed factors and their interactions on platelet-derived growth factor isoform BB (PDGF-BB) (model 3), and transforming growth factor beta-1 (TGF-β_1_) (model 4) concentrations (pg/mL).

GLMM Number	Fixed Effect	*p*-Value
	Intercept	<0.001
	Hemocomponent (HC)	<0.001
	Treatment	0.891
3	Time	0.994
	HC × Treatment	0.596
	HC × Time	0.215
	Treatment × Time	0.257
	HC × Treatment × Time	0.083
	Intercept	<0.001
	HC	<0.001
	Treatment	0.136
4	Time	0.917
	HC × Treatment	0.546
	HC × Time	0.058
	Treatment × Time	0.350
	HC × Treatment × Time	0.013

**Table 3:** GLMMs evaluating the effect of the fixed factors and their interactions on interleukin 1-beta (IL-1β, pg/mL) (model 5), and tumor necrosis factor-alpha (TNF-α, pg/mL) (model 6) release.

GLMM Number	Fixed Effect	*p*-Value
	Intercept	<0.001
	Hemocomponent (HC)	0.105
	Treatment	0.188
5	Time	0.576
	HC × Treatment	0.596
	HC × Time	0.019
	Treatment × Time	0.248
	HC × Treatment × Time	<0.001
	Intercept	<0.001
	HC	0.840
	Treatment	0.144
6	Time	0.884
	HC × Treatment	0.367
	HC × Time	0.553
	Treatment × Time	0.990
	HC × Treatment × Time	<0.001

**Table 4:** GLMM evaluating the effect of the fixed factors and their interactions on interleukin 10 (IL-10, pg/mL) release (model 7).

GLMM Number	Fixed Effect	*p*-Value
	Intercept	<0.001
	Hemocomponent (HC)	0.001
	Treatment	0.263
7	Time	<0.001
	HC × Treatment	0.046
	HC × Time	0.001
	Treatment × Time	0.066
	HC × Treatment × Time	<0.001

## Data Availability

The raw data supporting the conclusions of this article will be made available by the authors on request.
